# Racial Disparities in Treatment Initiation and Outcomes of Chronic Hepatitis B Virus Infection in North America

**DOI:** 10.1001/jamanetworkopen.2023.7018

**Published:** 2023-04-10

**Authors:** Mandana Khalili, Kelsey R. Leonard, Marc G. Ghany, Mohamed Hassan, Lewis R. Roberts, Richard K. Sterling, Steven H. Belle, Anna S. Lok, and the Hepatitis B Research Network

**Affiliations:** 1 Division of Gastroenterology and Hepatology, University of California, San Francisco; 2 Department of Epidemiology, School of Public Health, University of Pittsburgh, Pittsburgh, Pennsylvania; 3 Liver Diseases Branch, National Institute of Diabetes and Digestive and Kidney Diseases, National Institutes of Health, Bethesda, Maryland; 4 Division of Gastroenterology, Hepatology and Nutrition, University of Minnesota, Minneapolis; 5 Division of Gastroenterology and Hepatology, Mayo Clinic, Rochester, Minnesota; 6 Division of Gastroenterology, Hepatology, and Nutrition, Virginia Commonwealth University, Richmond; 7 Department of Biostatistics, School of Public Health, University of Pittsburgh, Pittsburgh, Pennsylvania; 8 Division of Gastroenterology and Hepatology, University of Michigan, Ann Arbor

## Abstract

**Importance:**

Disparities in treatment initiation may affect outcomes, but data on racially diverse populations with chronic hepatitis B virus (HBV) infection are limited.

**Objective:**

To examine whether HBV treatment initiation and outcomes differ among racial groups.

**Design, Setting, and Participants:**

From January 14, 2011, to January 28, 2018, hepatitis B surface antigen—positive adults (age ≥18 years) not receiving anti-HBV therapy were enrolled and followed up at weeks 12, 24, and every 24 weeks thereafter in a multicenter longitudinal cohort study (Hepatitis B Research Network [HBRN] adult cohort study) conducted in North America. The last study visit and data collection were completed January 28, 2019. Data were analyzed from August 27, 2021, to August 25, 2022. All HBRN participants were included unless they had acute HBV, HIV, hepatitis C or D, less than 24-weeks of follow-up after enrollment, initiated treatment at or immediately after enrollment, or had unknown race.

**Exposures:**

Participants had clinical and laboratory assessments and could receive anti-HBV treatment after enrollment.

**Main Outcomes and Measures:**

Hepatitis B virus treatment initiation and major adverse liver outcomes (hepatic decompensation, hepatocellular carcinoma, liver transplant, and death).

**Results:**

Of 1550 participants, 193 (12%) were African American or Black, 1157 (75%) were Asian, 157 (10%) were White, and 43 (3%) were other races; 789 (51%) were women, and the median age was 41.2 (IQR, 32.9-51.6) years. Sociodemographic and virologic parameters differed between groups. During 5727 person-years of follow-up, 504 participants initiated treatment, with incidences of 4.8 per 100 person-years in African American or Black individuals, 9.9 per 100 person-years in Asian individuals, 6.6 per 100 person-years in White individuals, and 7.9 per 100 person-years in those of other races (*P* < .001). A lower proportion (14%) of African American or Black participants met treatment criteria compared with Asian (22%) and White (27%) individuals (*P* = .01). The cumulative probabilities of treatment initiation after meeting the criteria were not significantly different among racial groups (African American or Black, 0.45; Asian, 0.38; White, 0.40 at 48 weeks and African American or Black, 0.45; Asian, 0.51; White, 0.51 at 72 weeks; *P* = .68). The incidence of major adverse liver outcomes was 0.1 per 100 person-years and did not differ by race.

**Conclusions and Relevance:**

In this observational study of chronic HBV, African American or Black participants were less likely than individuals of other races to meet treatment criteria, but among those who did, HBV treatment receipt did not differ significantly by race or socioeconomic factors. Not all eligible participants initiated treatment, but adverse liver outcomes were rare. These findings may not be generalizable to patients with chronic HBV receiving care in other settings.

## Key Points

**Question:**

Are there racial differences in initiating treatment and outcomes for chronic hepatitis B (CHB) in North American adults?

**Findings:**

In this cohort study of 1550 participants with CHB, during 5727 person-years of follow-up, the incidence of treatment initiation was 4.8 per 100 person-years in African American or Black individuals, 9.9 per 100 person-years in Asian individuals, 6.6 per 100 person-years in White individuals, and 7.9 per 100 person-years among those of other races. The cumulative percentage of treatment initiation for those meeting guideline-based criteria was 62% and was not significantly different among racial groups; major adverse liver outcomes were rare.

**Meaning:**

In this study, racial disparity in rates of treatment initiation among patients with CHB meeting criteria for treatment was not identified.

## Teaser

This cohort study examines treatment for chronic hepatitis B among a racially diverse group of individuals in North America.

## Introduction

There are racial disparities in the prevalence, outcomes, and access to treatment for individuals with chronic hepatitis B (CHB).^1,2^ The prevalence of CHB in the US is estimated at 2.4 million and disproportionately affects persons of Asian or African descent.^3^ Asian American and African American or Black individuals also have a higher incidence of hepatocellular carcinoma (HCC) and with development of HCC at an earlier age, compared with White persons with CHB.^4,5,6,7^ Whether racial discrepancies in outcomes are due to differences in the duration of infection, hepatitis B virus (HBV) genotypes, social determinants of health, or treatment access are unclear.

The Hepatitis B Research Network (HBRN) adult cohort study enrolled a racially diverse CHB cohort from 20 US centers and 1 center in Toronto, Canada.^8^ Earlier analyses of the baseline characteristics of the cohort found significant differences in the presumed mode of infection, HBV genotype, hepatitis B e antigen (HBeAg) status, HBV DNA levels, and alanine aminotransferase (ALT) levels across racial groups, and among African American or Black participants, significant differences between those born in the US, East Africa, and West Africa.^9^

We leveraged longitudinal data from the HBRN adult cohort study to examine whether treatment initiation and outcomes differed between African American or Black, Asian, and White participants and among African American or Black participants between those born in the US or Canada vs East or West Africa.

## Methods

### Study Design

The HBRN adult cohort study has been previously described.^8,10^ From January 14, 2011, to January 28, 2018, HBsAg-positive patients aged 18 years or older who were not receiving antiviral therapy unless pregnant or coinfected with hepatitis D and had no history of hepatic decompensation, HCC, or liver transplant were enrolled after providing written informed consent; financial compensation varied across study sites, guided by the local institutional review boards. The last study visit and data collection were completed January 28, 2019. The protocol was approved by the institutional review boards of each institution and by an NIDDK-appointed data and safety monitoring board. This report follows the Strengthening the Reporting of Observational Studies in Epidemiology (STROBE) reporting guideline.

Race and household income were self-reported, with race categorized herein as African American or Black, Asian, White, and other. Information on country of birth, duration of US or Canada residence, educational level, employment, insurance, prior antiviral treatment, family history of HBV or HCC, and mode of transmission were collected by research coordinators (eMethods in Supplement 1).

Participants had visits at baseline, weeks 12 and 24, and every 24 weeks thereafter with additional visits if they had ALT flare, pregnancy, or became HBeAg- or HBsAg-negative. Hepatocellular carcinoma surveillance followed the American Association for the Study of Liver Diseases (AASLD) guidelines.^11^ Participants could receive antiviral treatment as per standard of care or through HBRN treatment trials.^12,13^

Standard-of-care tests occurred at the local laboratories. Standardized cutoff values were chosen for the ALT upper limit of normal (ULN) level: 30 U/L for men and 20 U/L for women (to convert to microkatals per liter, multiply by 0.0167).

### Cohort Selection

All HBRN study participants were considered unless they met any of the following exclusion criteria: acute HBV, chronic HIV, chronic hepatitis C or D, follow-up less than 24 weeks, initiated treatment at enrollment or enrolled into an HBRN treatment trial immediately after enrollment, or their race was unknown (n = 3) (eFigure in Supplement 1). Baseline date was the date of enrollment. The HBsAg, HBeAg, HBV DNA, ALT, and platelet values on the same day or closest to each other during 24 weeks before to 18 weeks after enrollment were used as baseline laboratory values. Hepatitis B virus testing was performed as previously described.^8,10^ Of the 2032 participants, 1550 met the criteria for this secondary analysis.

### Treatment Initiation

Based on the timing of the HBRN study, we used the 2016 AASLD guidelines for treatment eligibility^11^ as follows: presence of cirrhosis or if no cirrhosis, HBV DNA level greater than 20 000 IU/mL and ALT level 2 × ULN or higher if HBeAg-positive, and HBV DNA level greater than 2000 IU/mL and ALT 2 × ULN or higher if HBeAg-negative. For this study, we focused on concordance of treatment initiation and treatment eligibility in all participants with cirrhosis (at or before enrollment or from the time of incident cirrhosis) and participants without cirrhosis who met treatment criteria on 2 consecutive visits to allow for a period of observation for spontaneous HBeAg loss or stability of HBV DNA and ALT levels and for health care professional–patient discussion of treatment plans. In a secondary analysis, participants with cirrhosis and all participants without cirrhosis who met treatment criteria at a single visit were included. Participants with less than 24 weeks of treatment (41% were pregnant) were not considered to have initiated treatment.

### Outcomes

All outcomes were predefined,^10^ and the occurrence and timing of ALT flares (ALT≥10 × ULN), incident cirrhosis, and adverse liver outcomes (hepatic decompensation, HCC, liver transplant, and death) were adjudicated by a committee of HBRN investigators. Loss of HBeAg and HBsAg was recorded.

### Statistical Analysis

Data analysis included all data collected up to last study visit on January 28, 2019. Data analyses began August 27, 2021, and were completed August 25, 2022. Participant characteristics are described by medians and quartiles for continuous variables and by frequencies and percentages for categorical variables. Statistical significance was tested with the Kruskal-Wallis, χ^2^, or Fisher exact test, as appropriate. Kaplan-Meier estimates for cumulative probabilities of treatment initiation were calculated by race and by region of origin among African American or Black participants and cumulative probabilities were compared using a 2-sided log-rank test. Crude incidence rates for outcomes per 100 person-years of follow-up were calculated. The Wald test was used for comparisons unless the number of events in a group was less than 5; if so, the Poisson exact test was used. Multivariable Cox proportional hazard models were used to evaluate the associations between the time to treatment initiation from meeting treatment indications with age, sex, HBV DNA (log_10_ IU/mL), ALT (log_10_ ULN), presence or absence of cirrhosis, race, educational level, income, insurance type, and time since immigration. Hepatitis B virus DNA level, ALT level, and presence or absence of cirrhosis were used as time-varying covariates, while the other covariates were fixed at baseline. The covariates for the multivariable model were selected a priori. Socioeconomic factors were included as possible confounders with race. Mode of transmission was considered but excluded due to substantial (29%) missing data. The minimum of time to treatment or loss to follow-up was used in the Cox proportional hazards model. Statistical analysis was conducted with SAS version 9.4 (SAS Institute Inc).

## Results

### Baseline Characteristics

Among 2032 HBRN-enrolled adults, 1550 met the criteria for this study (eFigure in Supplement 1); overall median age was 41.2 (IQR, 32.9-51.6) years, 789 participants (51%) were women and 761 were men (49%). A total of 193 (12%) participants were African American or Black, 1157 (75%) were Asian, 157 (10%) were White, and 43 (3%) were other races. Only 15 participants (<1%) reported Hispanic ethnicity. Given the small sample size, they were included in their respective racial group for analysis (9 White, 1 Black, 4 Asian, 1 other). There were statistically significant differences in sex, age, time since immigration to North America, HBV virologic characteristics, and mode of transmission and family history of HBV or HCC across the racial groups, but duration of follow-up was similar (Table 1). With respect to social determinants of health, African American or Black participants had a lower educational level, income, and proportion employed compared with other racial groups. Compared with White participants, a higher percentage of African American or Black and Asian participants were born outside North America and were uninsured or had public insurance. With respect to HBV disease characteristics, African American or Black participants had a lower prevalence of HBeAg and lower ALT levels than Asian individuals. Among HBeAg-positive participants, there were no statistically significant differences in HBV DNA levels across the racial groups, but among HBeAg-negative participants, African American or Black participants had lower HBV DNA levels than Asian participants.

**Table 1.** Baseline Characteristics of Participants by Race

Characteristic	No. (%)				*P* value
	African American or Black	Asian	White	Other^a^	
No.	193	1157	157	43	
Age at enrollment, median (IQR), y	41.1 (33.2-51.1)	40.8 (32.9-51.1)	48.1 (34.2-58.2)	38.1 (28.2-48.2)	.001
Sex					
Female	92 (48)	605 (52)	65 (41)	27 (63)	.02
Male	101 (52)	552 (48)	92 (59)	16 (37)	
Educational level					
No.	193	1147	154	43	
High school or equivalent or less	93 (48)	372 (32)	34 (22)	9 (21)	<.001
More than high school	100 (52)	775 (68)	120 (78)	34 (79)	
Total annual household income, $					
No.	149	921	134	37	
<25 000	73 (49)	244 (27)	22 (16)	11 (30)	<.001
25 000-49 999	35 (24)	191 (21)	31 (23)	7 (19)	
50 000-99 999	24 (16)	223 (24)	41 (31)	9 (24)	
≥100 000	17 (11)	263 (29)	40 (30)	10 (27)	
Employment status					
No.	193	1148	157	43	
Employed, full-time or part-time	123 (64)	888 (77)	118 (75)	29 (67)	<.001
Homemaker, not currently working for pay	5 (3)	64 (6)	4 (3)	3 (7)	
Not currently employed	65 (34)	196 (17)	35 (2)	11 (26)	
Type of insurance					
No.	189	1142	156	43	
None/self-pay	19 (10)	83 (7)	10 (6)	1 (2)	.003
Public/other	63 (33)	404 (35)	32 (21)	11 (26)	
Private	107 (57)	655 (57)	114 (73)	31 (72)	
Time since migration, y					
No.	180	1045	151	37	
NA (born in US or Canada)	39 (22)	108 (10)	97 (64)	22 (60)	
<10	66 (37)	282 (27)	9 (6)	2 (5)	<.001
10-20	50 (28)	277 (27)	28 (19)	6 (16)	
>20	25 (14)	378 (36)	17 (11)	7 (19)	
HBeAg status					
No.	174	1051	142	41	
Positive	21 (12)	319 (30)	21 (15)	12 (29)	<.001
HBV genotype					
No.	160	1082	136	42	
A1	67 (42)	44 (4)	3 (2)	1 (2)	<.001
A2	31 (19)	9 (1)	73 (54)	4 (10)	
A (other)	3 (2)	1 (0.1)	3 (2)	0 (0)	
B	7 (4)	544 (50)	7 (5)	5 (12)	
C	2 (1)	450 (42)	5 (4)	27 (64)	
D	16 (10)	32 (3)	41 (30)	5 (12)	
E	33 (21)	1 (0.1)	1 (1)	0 (0)	
Mixed/other	1 (1)	1 (0.1)	3 (2)	0 (0)	
Cirrhosis (before or at enrollment)	2 (1)	8 (1)	9 (6)	0 (0)	<.001
HBsAg, median (IQR), log_10_ IU/mL	3.8 (3.2-4.3)	3.3 (2.6-4.0)	3.8 (2.9-4.3)	3.9 (3.3-4.3)	<.001
No.	168	1016	137	39	
HBV DNA, median (IQR), log_10_ IU/mL					
No.	183	1085	150	42	
Overall	3.1 (2.1-4.1)	4.1 (2.8-6.3)	3.1 (2.1-4.5)	3.7 (2.6-5.4)	<.001
HBeAg+	8.1 (6.8-8.4)	8.0 (6.2-8.3)	8.3 (7.9-8.7)	8.3 (7.7-8.6)	.06
HBeAg−	2.9 (2.0-3.6)	3.3 (2.5-4.6)	2.8 (2.1-3.6)	2.9 (2.1-3.8)	<.001
ALT, median (IQR), U/L^b^	29.0 (20.5-52.0)	34.0 (23.0-53.0)	37.0 (25.0-57.0)	32.5 (20.0-54.5)	.19
No.	132	691	97	28	
Platelets, median (IQR), ×10^3^/μL	204.0 (169.0-261.0)	216.0 (178.0-251.5)	221.0 (182.0-258.5)	217.0 (184.0-265.0)	.70
No.	117	576	80	26	
Presumed mode of HBV transmission					
No.	140	877	101	34	
Vertical	29 (21)	618 (71)	25 (25)	30 (88)	<.001
Horizontal	111 (79)	259 (30)	75 (74)	4 (12)	
Other	0 (0)	0 (0.0)	1 (1)	0 (0)	
HBV infection in family members					
No.	136	947	122	34	<.001
Yes	41 (30)	691 (73)	36 (30)	27 (79)	
Liver cancer in family members					
No.	170	1025	134	39	<.001
Yes	9 (5)	204 (20)	7 (5)	7 (18)	
HBV treatment before enrollment	9 (5)	204 (20)	7 (5)	7 (18)	.05
Weeks of follow-up, median (IQR)	257.0 (118.0-345.6)	269.4 (138.3-359.9)	302.9 (164.1-363.1)	266.4 (117.1-358.0)	.11

Abbreviations: ALT, alanine aminotransferase; HBeAg, hepatitis B e antigen; HBsAg, hepatitis B s antigen; HBV, hepatitis B virus; NA, not applicable;−, negative; +, positive.

SI conversion: to convert ALT to microkatals per liter, multiply by 0.0167; platelets to ×10^9^ per liter, multiply by 1.

^a^ Other includes Indian, Iranian, Kazakhstani, Middle Eastern, Uzbekistani, and mixed.

^b^ The upper limit of normal for ALT levels was 30 U/L for men and 20 U/L for women.

Of the 193 African American or Black participants, 39 (20%) were born in the US, 90 (47%) in East Africa, 53 (27%) in West Africa, and 11 (6%) elsewhere. Baseline characteristics by birth region differed significantly with respect to age, HBV characteristics, and measures of socioeconomic status (eTable 1 in Supplement 1). Compared with those born in the US, Africa-born African American or Black participants were younger and had a lower prevalence of HBeAg and genotype A2.

### Treatment Initiation

#### Overall

**Figure 1.**
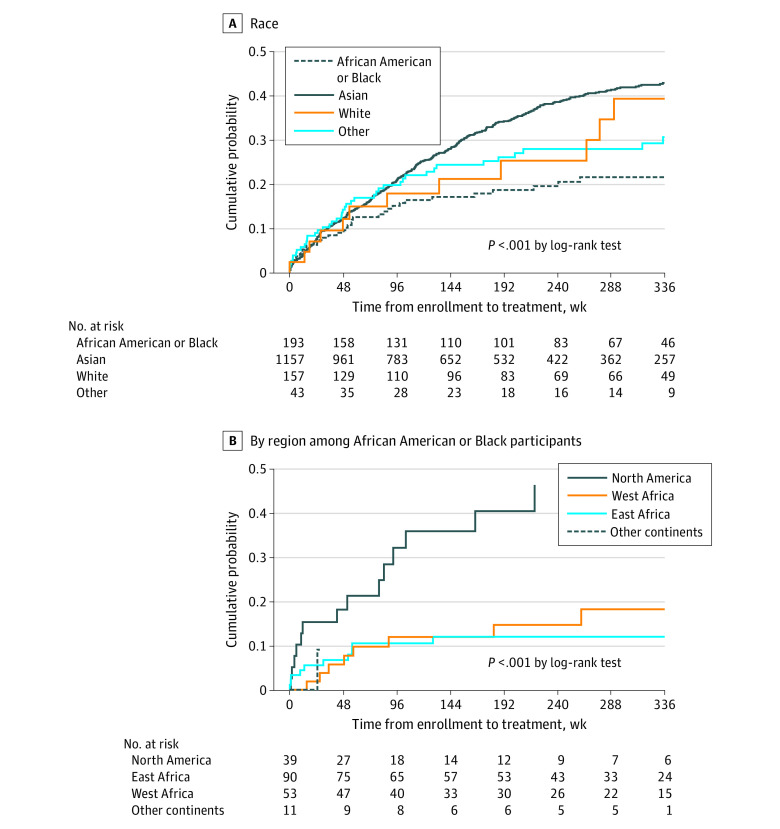
Cumulative Probability of Treatment Initiation From Enrollment A, Cumulative probability of treatment initiation from enrollment by race. B, Cumulative probability of treatment initiation from enrollment by region of origin among African American or Black participants.

During 5727 person-years of follow-up, 504 (35 African American or Black, 415 Asian, 42 White, and 12 of other races) participants initiated antiviral treatment, with incidence of treatment initiation per 100 person-years of 4.8 among African American or Black, 9.9 among Asian, and 6.6 among White individuals, and 7.9 among those of other races (*P* < .001). Among African American or Black participants, there were significant differences in the incidence of treatment initiation per 100 person-years, being 14.3 for those born in the US or Canada, 2.8 for East Africa, 3.6 for West Africa, and 4.8 for other regions (*P* < .001). Asian participants had the highest cumulative probability and African American or Black participants had the lowest probability of treatment initiation (Figure 1A). Among African American or Black participants, those born in the US had a higher probability of treatment initiation than those born elsewhere (Figure 1B).

#### Participants Who Met 2016 AASLD Treatment Criteria

During the study, a lower percentage of African American or Black participants (14%) met treatment criteria compared with Asian (22%) and White (27%) participants (*P* = .01). This difference remained when the analysis included participants without cirrhosis who met treatment criteria only once.

**Figure 2.**
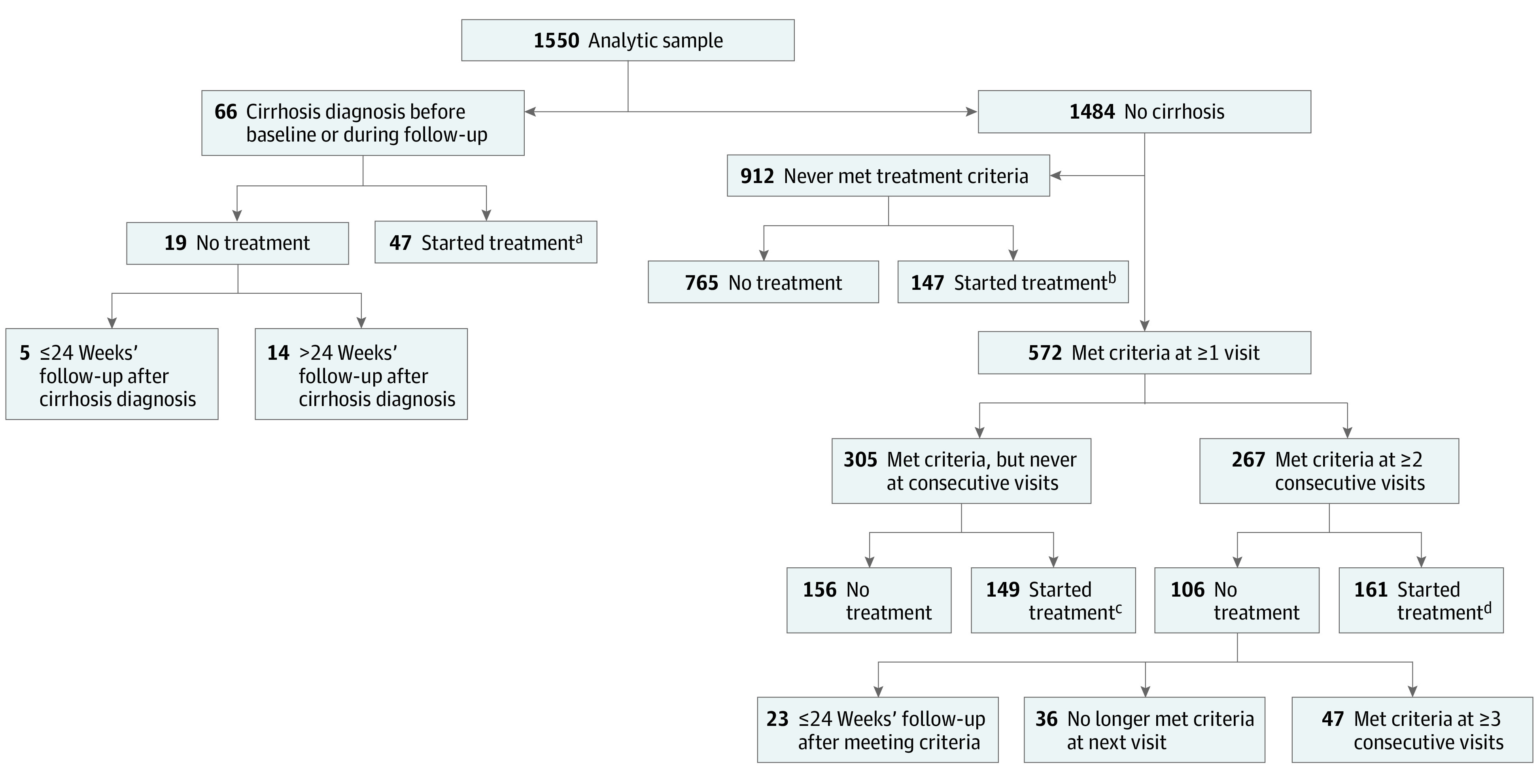
Participants Meeting Treatment Criteria and Initiating Treatment During Follow-up ^a^Three participants initiated treatment through the Hepatitis B Research Network (HBRN) treatment trials. ^b^Twenty-four participants initiated treatment through the HBRN treatment trials. ^c^Twenty-eight participants initiated treatment through the HBRN treatment trials. ^d^Fifty-seven participants initiated treatment through the HBRN treatment trials.

A total of 66 participants had cirrhosis (19 at enrollment and 47 during follow-up); of these, 47 (71%) started treatment, 5 (11%) had less than 24 weeks’ follow-up after cirrhosis diagnosis, and 14 (30%) never started treatment through the end of the study (Figure 2). Longitudinal data of the 14 participants who did not start treatment showed 5 had persistently and 2 had intermittently unquantifiable HBV DNA levels, and 1 individual had a single HBV DNA level at 30 IU/mL without further follow-up.

Among participants who met criteria once and did not immediately start treatment, less than half (47%) met criteria at the next visit, and for those who met criteria on 2 consecutive visits and had not started treatment, more than half (56%) no longer met criteria at the third visit. Of 1484 participants who did not have cirrhosis at enrollment or during follow-up, 572 (39%) met treatment criteria at least once and 267 (18%) met the criteria on 2 or more consecutive visits, of whom 161 (60%) started treatment (Figure 2). The cumulative probability of treatment initiation by 144 weeks after meeting the criteria was 0.64, with most participants initiating treatment by 48 weeks. Of the 106 participants who did not start treatment after meeting treatment criteria on 2 or more consecutive visits, 36 (34%) no longer met the criteria at their third visit and 23 (22%) had no further follow-up. Compared with the 161 patients who met treatment criteria on 2 or more consecutive visits and initiated treatment, a higher percentage of the 47 patients who still met the criteria at the third visit but were not treated were women (70% vs 49%), had a higher annual household income (≥$50 000: 64% vs 40%), a higher prevalence of family history of HBV (80% vs 59%), and a presumed vertical mode of transmission (81% vs 61%) (eTable 2 in Supplement 1).

Higher ALT levels, HBV DNA levels, and presence of cirrhosis were associated with a significantly shorter time to treatment initiation. When adjusting for disease factors, neither race nor socioeconomic factors were associated with time to treatment initiation (Table 2).

**Table 2.** Multivariable Model Evaluating the Associations Between the Time to Treatment Initiation From Meeting AASLD Treatment Criteria^a^

Covariate	HR (95% CI)	*P* value
ALT, log_10_ × ULN^b^	6.76 (3.91-11.68)	<.001
HBV DNA, log_10_ IU/mL^b^	1.17 (1.04-1.32)	.01
Cirrhosis^b^		
Yes	2.84 (1.51-5.34)	.001
No	1 [Reference]	
Race		.82
Asian	1 [Reference]	
African American or Black	0.74 (0.34-1.65)	
White	1.19 (0.60-2.35)	
Other^c^	1.11 (0.23-5.34)	
Age, y	1.02 (1.00-1.04)	.04
Sex		
Male	1 [Reference]	.14
Female	0.75 (0.51-1.11)	
Educational level		
High school equivalent or less	0.84 (0.55-1.28)	.42
More than high school	1 [Reference]	
Annual household income, $		
≥50 000	1 [Reference]	.31
<25 000	1.56 (0.88-2.75)	
25 000 to <50 000	1.32 (0.76-2.30)	
Insurance		
Private	1 [Reference]	.44
None/self-pay	1.21 (0.65-2.25)	
Public/other	1.31 (0.86-1.99)	
Years since migration to US or Canada, y		
Born in US or Canada	1 [Reference]	.72
<10	1.11 (0.57-2.18)	
10-20	1.31 (0.70-2.44)	
>20	0.98 (0.50-1.89)	

Abbreviations: AASLD, American Association for the Study of Liver Diseases; ALT, alanine aminotransferase; HBV, hepatitis B virus; HR, hazard ratio; ULN, upper limit of normal.

^a^ Of 1550 participants, 333 met criteria for treatment (once for those with cirrhosis and on 2 consecutive visits for those who did not have cirrhosis). A total of 123 participants were excluded: 22 for starting treatment before meeting criteria, 20 with cirrhosis for missing ALT or HBV DNA levels within 24 weeks of cirrhosis diagnosis, and 81 due to missing data for educational level, income, insurance, and/or time since migration. Thus, 210 participants were included in this analysis, of whom 125 initiated treatment.

^b^ Time-dependent covariates, ie, these variables changed during follow-up.

^c^ Other includes Indian, Iranian, Kazakhstani, Middle Eastern, Uzbekistani, and mixed.

Among the 305 participants who met treatment criteria at least once but never at consecutive visits, 149 (49%) started treatment (Figure 2). Compared with participants who did not start treatment, those who did were older (median, 42.2 [IQR, 33.4-53.1] vs 38.4 [IQR, 32.1-45.3] years), more likely to be male (80 [54%] vs 65 [42%]), and HBeAg-positive (61 [46%] vs 48 [33%]), and had higher median HBV DNA (5.7 vs 4.8 log IU/mL) and ALT (59 vs 34 U/L) levels, but there were no significant differences in race or socioeconomic factors.

**Figure 3.**
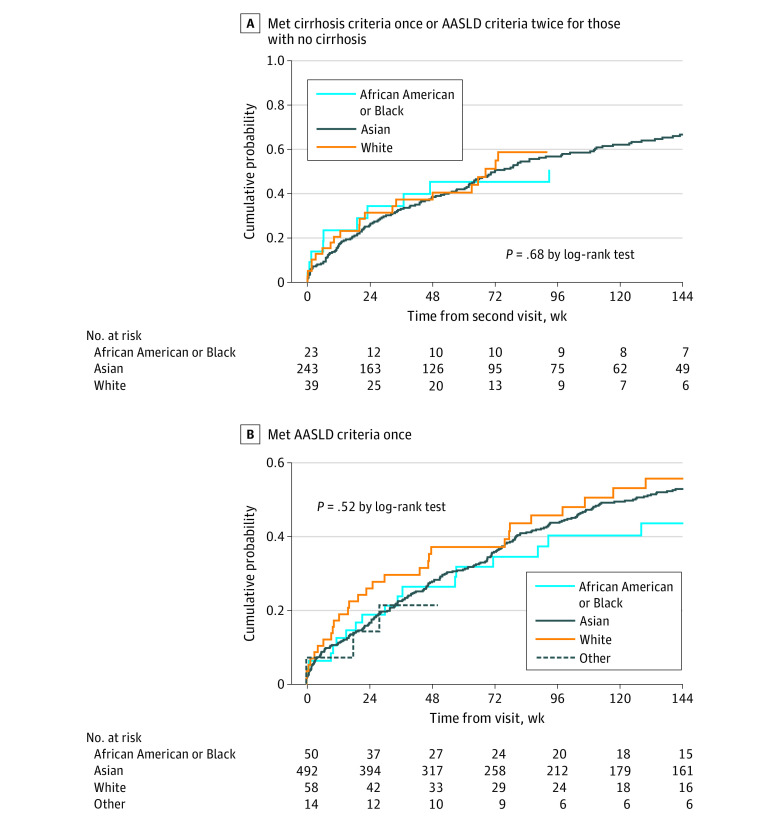
Cumulative Probability of Treatment Initiation From Meeting Treatment Criteria by Race A, Cumulative probability of treatment initiation from meeting treatment criteria defined as meeting cirrhosis criteria once or meeting American Association for the Study of Liver Diseases (AASLD) treatment criteria for participants without cirrhosis on 2 visits. Other race is not shown because the number at risk was too small to provide a reliable estimate. B, Cumulative probability of treatment initiation from meeting treatment criteria defined as meeting AASLD treatment criteria once.

Figure 3A shows cumulative probabilities of treatment initiation after meeting criteria for cirrhosis once or after meeting AASLD treatment criteria for participants without cirrhosis on 2 consecutive visits across racial groups (African American or Black, 0.45; Asian, 0.38; White, 0.40 at 48 weeks and African American or Black, 0.45; Asian, 0.51; White, 0.51 at week 72; *P* = .68). Such differences remained when the analysis included participants meeting AASLD treatment criteria once (Figure 3B). Similarly, among African American or Black participants who met treatment criteria, there were no significant differences in cumulative probabilities of treatment by region of birth.

#### Participants Who Never Met AASLD Treatment Criteria

Of 912 participants without cirrhosis and who never met AASLD treatment criteria, 147 started treatment. These individuals were older, more likely to be HBeAg-positive, and had higher HBV DNA and ALT levels than those who did not start treatment (eTable 3 in Supplement 1).

### Outcomes

Adverse liver outcomes were rare, with an overall incidence of 0.1 per 100 person-years for the composite outcome of hepatic decompensation, HCC, liver transplant, or HBV-related death (eTable 4 in Supplement 1). During follow-up, 7 cases of HCC (0 African American or Black, 5 Asian, and 2 White participants; 4 receiving HBV therapy), 2 cases of hepatic decompensations (1 African American or Black and 1 White participant), and 47 cases of incident cirrhosis (8 African American or Black, 28 Asian, 9 White, and 2 other race participants; 14 receiving HBV therapy) occurred. In addition, 93 participants experienced at least 1 ALT flare, 90 lost HBsAg, and 112 of 363 HBeAg-positive participants lost HBeAg. There were no significant differences in these outcomes by race except for a lower rate of HBsAg loss among Asian vs African American or Black and White participants (eTable 4 in Supplement 1).

A similar percentage of participants who did (n = 161) vs did not (n = 47) initiate therapy after meeting the criteria on 2 or more consecutive visits experienced ALT flares, HBeAg loss, or HBsAg loss. None of these 208 participants had incident cirrhosis, hepatic decompensation, HCC, liver transplant, or HBV-related death.

## Discussion

In this CHB cohort, rates of treatment initiation were highest among Asian and lowest among African American or Black participants corresponding to differences in meeting treatment criteria. Among participants with a treatment indication, treatment rates did not differ significantly by race, despite marked differences in educational level, income, and type of health care insurance across the racial groups. Moreover, race was not an independent estimator of treatment initiation when adjusting for known factors associated with a higher risk of adverse clinical outcomes, namely, HBV DNA, disease severity, sex, and age.

Patient and health care professional knowledge and attitudes are important to HBV treatment initiation.^14,15,16,17,18^ Barriers in accessing care are also a major impediment to equitable health care and clinical outcomes.^19,20^ In this study, all participants were linked to liver specialists who may have enhanced treatment rates^21^ or had access to HBRN treatment trials if eligible, contributing to the lack of differences in treatment initiation rates by race among those meeting treatment criteria. Although the HBRN study was not designed to evaluate determinants of initiating treatment, we were able to examine some socioeconomic factors. Differences in educational level, household income, types of health care insurance, and duration of North American residence had no association with treatment initiation among those who met the criteria. However, the influence of socioeconomic factors may have been underestimated due to limited data. Similar to our study, in a recent retrospective analysis of a US national database of more than 12 000 patients with CHB with private insurance, neither race nor social determinants of health were associated with treatment initiation among eligible patients.^21^

There are likely many reasons contributing to the lack of treatment initiation among eligible participants. Given the dynamic nature of CHB, some participants meeting treatment criteria at 1 visit may no longer qualify for treatment at the subsequent visit. Indeed, among participants who met criteria once and did not immediately start treatment, less than half (47%) met criteria at the next visit, and for those who met criteria on 2 consecutive visits and had not started treatment, more than half (56%) no longer met criteria at the third visit. Additionally, rather than initiating treatment based on a single set of laboratory values, some clinicians and participants may have opted to monitor for a longer period before committing to therapy that may be lifelong. Among participants who met the treatment criteria, key factors associated with initiating treatment were sex, mode of transmission, and family history of HBV infection. In this analysis, women had lower treatment initiation rates compared with men, which is similar to other reports.^22^ This may relate to sex differences in acceptance of treatment or patient and/or clinician perception of lower risk of adverse outcomes in women. Lower treatment initiation based on a family history of HBV may relate to stigma associated with treatment.^23^ Moreover, 1 study found that patients misinterpret having a family history to mean that CHB is an inherited disease and treatment would not prevent outcomes.^24^

Current treatment for CHB is effective in suppressing HBV replication and preventing adverse outcomes; however, these treatments are not curative.^25^ Professional societies have established guideline criteria for treatment largely based on cirrhosis, HBV DNA levels, and ALT levels.^26,27,28^ This study noted that African American or Black participants had a lower prevalence of HBeAg and lower HBV DNA levels among those who were HBeAg-negative, leading to a lower percentage meeting treatment criteria. An important concern raised by some experts is whether thresholds for starting therapy should be lower in African American or Black individuals compared with those of other races.^29^ This viewpoint is based on studies showing HBV-related complications—notably HCC—occurring at a younger age and low HBV DNA levels among persons living in Africa.^2,30,31,32^ In this study, HCC was not observed in the African-born African American or Black participants, including 125 not receiving treatment during the median follow-up of 5 years. This study cannot address whether the threshold for treatment initiation should be tailored to race or country of birth, but it raises the question whether environmental factors (eg, exposure to carcinogens such as aflatoxin) may contribute to early HCC among persons living in Africa.

Asian individuals with CHB also have a higher incidence of HCC and earlier occurrence.^33^ In this study, Asian participants had the highest prevalence of HBeAg and the highest percentage meeting treatment criteria and were most likely to initiate treatment; however, treatment initiation among those eligible was comparable to that of African American or Black and White participants, and clinical outcomes including HCC were also similar.

### Limitations

This study has limitations. Participants in the HBRN study were linked to specialty liver clinics and the findings may not be generalizable to other CHB patients in North America receiving care in other settings. Changes in guideline recommendations and standard-of-care practice may have influenced treatment decisions. Treatment initiation rates may also have changed with increased availability of generic nucleos(t)ide analogues in recent years. Additionally, the reasons for lack of treatment initiation in eligible patients are complex and many unmeasured factors, such as health literacy, patient preference, and health care professional perspective, may have played a role in treatment decisions, but these data were not collected. All variables in the multivariable model were selected a priori, many because we considered them to be confounders for race (eg, socioeconomic status), and others because we hypothesized that they were associated with treatment initiation. Future studies that span patient-, health care professional–, and system-level factors and levels of influence (individual, interpersonal, community, and societal) within these domains are needed to address the multifaceted nature of disparities in HBV care. While the treatment rates may not be optimal in the HBRN study, they are higher than earlier reports of less than 30%.^34,35^ It is also reassuring that the incidence of hepatic decompensation and HCC was very low in the HBRN study,^10^ with no significant difference across the racial groups when patients are closely monitored.

## Conclusions

In this large multiracial CHB cohort in North America, race and socioeconomic factors were not associated with treatment initiation. We observed a treatment gap between participants eligible for and those receiving treatment, suggesting that efforts to increase awareness of HBV and training of health care professionals, along with simplifying treatment guidelines, will be necessary to achieve the World Health Organization’s goals of HBV elimination by 2030.^36^

## Notes

Supplement 1. **eMethods.** Detailed Methods **eFigure.** Flow Diagram Showing Participant Selection **eTable 1.** Baseline Characteristics of African American/Black Participants by Region of Birth **eTable 2.** Baseline Characteristics of Participants Meeting AASLD Treatment Criteria on 2 Consecutive Visits, Who Did and Did Not Initiate Treatment **eTable 3.** Baseline Characteristics of Participants Who Never Met Criteria, and Did Versus Did Not Initiate Treatment **eTable 4.** Incidence of Adverse Liver Outcomes Overall and by Race

Supplement 2. **Nonauthor Collaborators.** The Hepatitis B Research Network (HBRN)

Supplement 3.
Data Sharing Statement
