# Supplementary material for: Racial Disparities in Treatment Initiation and Outcomes of Chronic Hepatitis B Virus Infection in North America
Source: JAMA Netw Open. 2023 Apr 10;6(4):e237018. doi: 10.1001/jamanetworkopen.2023.7018 (PMC10087055; doi:10.1001/jamanetworkopen.2023.7018)
Supplement: Supplement 1. — eMethods. Detailed Methods eFigure. Flow Diagram Showing Participant Selection eTable 1. Baseline Characteristics of African American/Black Participants by Region of Birth eTable 2. Baseline Characteristics of Participants Meeting AASLD Treatment Criteria on 2 Consecutive Visits, Who Did and Did Not Initiate Treatment eTable 3. Baseline Characteristics of Participants Who Never Met Criteria, and Did Versus Did Not Initiate Treatment eTable 4. Incidence of Adverse Liver Outcomes Overall and by Race [file jamanetwopen-e237018-s001.pdf]

Supplementary Online Content

Khalili M, Leonard KR, Ghany MG, et al; Hepatitis B Research Network. Racial disparities in treatment initiation and outcomes of chronic hepatitis B virus infection in North America. *JAMA Netw Open*. 2023;6(4):e237018. doi:10.1001/jamanetworkopen.2023.7018

eMethods. Detailed Methods

eFigure. Flow Diagram Showing Participant Selection

eTable 1. Baseline Characteristics of African American/Black Participants by Region of Birth

eTable 2. Baseline Characteristics of Participants Meeting AASLD Treatment Criteria on 2 Consecutive Visits, Who Did and Did Not Initiate Treatment

eTable 3. Baseline Characteristics of Participants Who Never Met Criteria, and Did Versus Did Not Initiate Treatment

eTable 4. Incidence of Adverse Liver Outcomes Overall and by Race

This supplementary material has been provided by the authors to give readers additional information about their work.

© 2023 Khalili M et al. *JAMA Network Open*.

eMethods. Detailed Methods

HBRN Adult Cohort description and sample size calculation:

A total of 2032 adults were enrolled in the cohort between January 2011 and January 2018. Participants were followed from enrollment through January 2019. As an observational cohort study, original sample size calculations were derived for two examples of analyses to provide an idea of the number of participants that would be required to test certain hypotheses with 80% power and 5% type I error using a two-sided test using covariate and outcome distributions obtained from the literature, surveys of the clinical centers, or that covered a wide range. However, recruitment lagged behind expectations based on those figures, so after 1416 participants were recruited, we recalculated power using actual data from the enrolled cohort and sample sizes of 1500 and 2500. Based on those results, the Steering Committee and external Data and Safety Monitoring Board approved reducing the sample size to 2000. Potential participants who were in the screening "pipeline" were enrolled so the final sample size was 2032.

Definition and categorization of variables:

Race/ethnicity [AA/B, Asian, White, other (Mixed, Indian, Iranian, Kazakhstanian, Middle Eastern, Uzbekistan)] and household annual income (<$25000, $25000-$49999, $50000-$99999, and ≥$100000) were self-reported. Information on country of birth, duration of residence in U.S. or Canada (born in North America, <10 years, 10-20 years, >20 years), education level (at most high school or equivalent, more than high school or equivalent), employment (employed full- or part-time, homemaker not for pay, not currently employed), type of healthcare insurance (none/self-pay, public/other, private), prior antiviral treatment (yes, no), family history of HBV or HCC (yes, no), and mode of transmission (vertical, horizontal, other) were collected by research coordinators. No other quantitative variables were categorized.

© 2023 Khalili M et al. *JAMA Network Open*.

eFigure 1 – Flow diagram showing participant selection

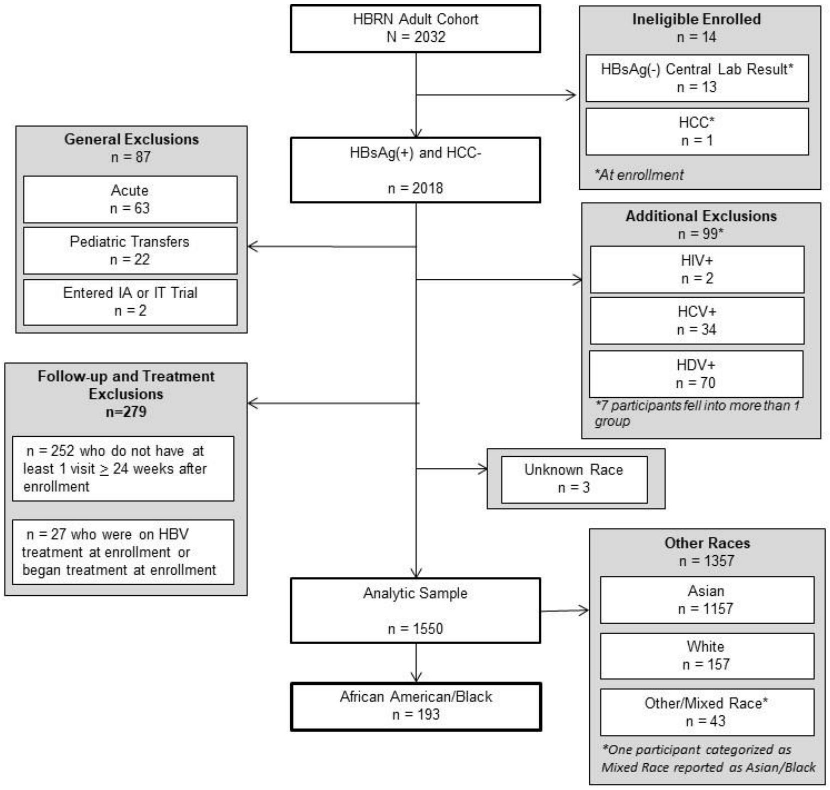

© 2023 Khalili M et al. *JAMA Network Open*.

eTable 1. Baseline characteristics of African American/Black participants by region of birth

**Characteristics**	**USA n=39**	**East Africa n=90**	**West Africa n=53**	**Other continents n=11**	*p-value*
**Age at Enrollment (Years)**	47.7 (40.5 : 58.7)	40.6 (32.8 : 50.8)	37.9 (30.9 : 44.8)	54.4 (40.4 : 61.3)	0.001
**Sex**: Female	21 (54%)	49 (54%)	18 (34%)	4 (36%)	0.08
**Education Level**	n=39	n=90	n=53	n=11	<0.001
High school or equivalent or less	20 (51%)	59 (66%)	11 (21%)	3 (27%)	
More than high school	19 (49%)	31 (34%)	42 (79%)	8 (73%)	
**Total Annual Household Income**[c]	n=32	n=62	n=48	n=7	<0.001
Less than $25,000	14 (44%)	42 (68%)	15 (31%)	2 (29%)	
$25,000 - $49,999	7 (22%)	13 (21%)	13 (27%)	2 (29%)	
$50,000 - $99,999	9 (28%)	5 (8%)	7 (15%)	3 (43%)	
$More than $100,000	2 (6%)	2 (3%)	13 (27%)	0 (0%)	
**Employment status**	n=39	n=90	n=53	n=11	0.24
Employed, full-time or part-time	23 (59%)	52 (58%)	39 (74%)	9 (82%)	
Homemaker, not currently working for pay	0 (0%)	3 (3%)	2 (4%)	0 (0%)	
Not currently employed	16 (41%)	35 (39%)	12 (23%)	2 (18%)	
**Type of Insurance**		n=87	n=52		0.11
None/Self Pay	5 (13%)	4 (5%)	8 (15%)	2 (18%)	
Public/Other	15 (39%)	27 (31%)	15 (29%)	6 (55%)	
Private	19 (49%)	56 (64%)	29 (56%)	3 (27%)	
**HBeAg Status**	n=36	n=80	n=48	n=10	0.11
Positive	8 (22%)	6 (8%)	5 (10%)	2 (20%)	
**HBV Genotype**	n=29	n=76	n=46	n=9	<0.001
A1	0 (0%)	57 (75%)	3 (7%)	7 (78%)	

© 2023 Khalili M et al. *JAMA Network Open*.

**Characteristics**	**USA n=39**	**East Africa n=90**	**West Africa n=53**	**Other continents n=11**	*p-value*
A2	26 (90%)	1 (1%)	3 (7%)	1 (11%)	
A(Other)	0 (0%)	0 (0%)	3 (7%)	0 (0%)	
B	2 (7%)	1 (1%)	3 (7%)	1 (11%)	
C	1 (3%)	1 (1%)	0 (0%)	0 (0%)	
D	0 (0%)	14 (18%)	2 (4%)	0 (0%)	
E	0 (0%)	2 (3%)	31 (67%)	0 (0%)	
Other[b]	0 (0%)	0 (0%)	1 (2%)	0 (0%)	
**Cirrhosis (prior to or at enrollment)**	2 (5.1%)	0 (0%)	0 (0%)	0 (0%)	0.10
**HBsAg (log_{10} IU/mL)**	n=35	n=77	n=46	n=10	0.11
	3.7 (2.5 : 4.5)	3.7 (3.1 : 4.1)	4.1 (3.6 : 4.3)	3.4 (3.2 : 4.4)	
**HBV DNA (log_{10} IU/mL)**	n=37	n=87	n=49	n=10	
Overall	3.4 (2.0 : 5.4)	2.9 (2.1 : 3.6)	3.4 (2.2 : 4.2)	3.5 (2.6 : 4.0)	0.24
HBeAg+	8.5 (7.9 : 8.9)	6.6 (5.4 : 7.7)	8.1 (8.1 : 8.1)	7.1 (5.8 : 8.4)	0.02
HBeAg-	2.9 (1.7 : 3.7)	2.7 (2.0 : 3.3)	3.1 (2.0 : 4.0)	3.3 (2.3 : 3.6)	0.44
**ALT (U/L)**	n=22	n=61	n=41	n=8	0.01
	29.0 (20.0 : 63.0)	25.0 (18.0 : 37.0)	43.0 (27.0 : 69.0)	27.0 (21.0 : 44.5)	
**Platelets ($x10^3/\mu L$)**	n=19	n=54	n=37	n=7	0.04
	226.0 (182.0 : 303.0)	185.0 (159.0 : 244.0)	208.0 (171.0 : 263.0)	255.0 (231.0 : 293.0)	
**Presumed mode of HBV transmission**	n=21	n=77	n=34	n=8	0.002
Vertical	2 (10%)	11 (14%)	15 (44%)	1 (13%)	
Horizontal	19 (91%)	66 (86%)	19 (56%)	7 (88%)	
**HBV Infection in Family Members**	n=33	n=60	n=36	n=7	0.13

© 2023 Khalili M et al. *JAMA Network Open*.

Characteristics	USA<br>n=39	East Africa<br>n=90	West Africa<br>n=53	Other continents<br>n=11	*p-value*
Yes	7 (21%)	17 (28%)	16 (44%)	1 (14%)	
**Liver Cancer in Family Members**	n=35	n=79	n=47	n=9	0.35
Yes	3 (9%)	2 (3%)	4 (9%)	0 (0%)	
**HBV treatment prior to enrollment**					0.01
Yes	7 (18%)	2 (2%)	4 (8%)	2 (18%)	
**Time since migration**		n=83	n=47		<0.001
N/A: born in US/Canada	39 (100%)	0 (0%)	0 (0%)	0 (0%)	
<10 years	0 (0%)	47 (57%)	17 (36%)	2 (18%)	
10-20 years	0 (0%)	31 (37%)	18 (38%)	1 (9%)	
>20 years	0 (0%)	5 (6%)	12 (26%)	8 (73%)	
**Weeks of follow-up**	261.0 (68.0 : 364.0)	257.0 (124.4 : 342.0)	255.1 (118.0 : 357.0)	291.3 (51.9 : 334.0)	0.97

[a] continuous data presented as median (25th : 75th percentile)

[b] Other race include: Mixed, Indian, Iranian, Kazakhstanian, Middle Eastern, Uzbekistan

© 2023 Khalili M et al. *JAMA Network Open*.

eTable 2. Baseline characteristics of participants meeting AASLD treatment criteria on two consecutive visits, who did and did not initiate treatment

**Characteristics**	**Met AASLD criteria at consecutive visits and started treatment n=161**	**Met AASLD criteria at consecutive visits did not start treatment n=47**	*p-value*
**Age at Enrollment (Years)**	38.3 (29.7 : 44.9)	36.6 (29.3 : 49.9)	0.58
**Sex**, female	79 (49%)	33 (70%)	0.011
**Race**	n=161	n=47	0.70
White	14 (9%)	5 (11%)	
African American/Black	8 (5%)	1 (2%)	
Asian	137 (85%)	41 (87%)	
Other[b]	2 (1%)	0 (0%)	
**Education Level**	n=161	n=47	0.06
High school or equivalent or less	62 (39%)	11 (23%)	
More than high school	99 (62%)	36 (77%)	
**Total Annual Household Income**	n=123	n=36	0.045
Less than $25,000	49 (40%)	11 (31%)	
$25,000 - $49,999	25 (20%)	2 (6%)	
$50,000 - $99,999	27 (22%)	14 (39%)	
$More than $100,000	22 (18%)	9 (25%)	
**Employment status**	n=161	n=47	0.45
Employed, full-time or part-time	131 (81%)	34 (72%)	
Homemaker, not currently working for pay	4 (3%)	2 (4%)	
Not currently employed	26 (16%)	11 (23%)	
**Type of Insurance**	n=160	n=47	0.86
None/Self Pay	17 (11%)	4 (9%)	

© 2023 Khalili M et al. *JAMA Network Open*.

Characteristics	Met AASLD criteria at consecutive visits and started treatment n=161	Met AASLD criteria at consecutive visits did not start treatment n=47	*p-value*
Public/Other	72 (45%)	20 (43%)	
Private	71 (44%)	23 (49%)	
**HBeAg Status**	n=148	n=46	0.23
Positive	92 (62%)	24 (52%)	
**HBV Genotype**	n=161	n=47	0.85
A1	6 (4%)	2 (4%)	
A2	9 (6%)	5 (11%)	
B	68 (42%)	19 (40%)	
C	67 (42%)	19 (40%)	
D	8 (5%)	1 (2%)	
E	3 (2%)	1 (2%)	
**HBsAg (log_{10} IU/mL)**	n=139	n=46	0.30
	4.0 (3.2 : 4.5)	4.2 (3.4 : 4.5)	
**HBV DNA (log_{10} IU/mL)**	n=151	n=47	
Overall	7.0 (5.3 : 8.2)	6.7 (5.1 : 8.2)	0.89
HBeAg+	8.1 (7.1 : 8.4)	8.2 (8.0 : 8.4)	0.13
HBeAg-	5.0 (3.9 : 5.7)	5.1 (4.6 : 5.5)	0.64
**ALT (U/L)**	n=112	n=37	0.28
	66.0 (41.0 : 104.5)	55.0 (36.0 : 88.0)	
**Platelets (x10^3/μL)**	n=88	n=29	0.09
	202.5 (165.0 : 248.5)	239.0 (192.0 : 259.0)	
**Presumed mode of HBV transmission**	n=118	n=32	0.03

© 2023 Khalili M et al. *JAMA Network Open*.

**Characteristics**	**Met AASLD criteria at consecutive visits and started treatment n=161**	**Met AASLD criteria at consecutive visits did not start treatment n=47**	*p-value*
Vertical	72 (61%)	26 (81%)	
Horizontal	46 (39%)	6 (19%)	
**HBV Infection in Family Members**	n=127	n=40	0.02
Yes	75 (59%)	32 (80%)	
**Liver Cancer in Family Members**	n=138	n=44	0.14
Yes	16 (12%)	9 (21%)	
**HBV treatment prior to enrollment**	n=161	n=47	0.06
Yes	35 (22%)	4 (9%)	
**Years since migration**	n=153	n=45	0.40
N/A: born in US/Canada	22 (14%)	8 (18%)	
<10 years	50 (33%)	10 (22%)	
10-20 years	48 (31%)	13 (29%)	
>20 years	33 (22%)	14 (31%)	
**Weeks of follow-up**	n=161	n=47	0.026
	263.3 (102.1 : 360.3)	336.4 (189.7 : 379.3)	

[a] ULN for ALT was 30 U/L for males and 20 U/L for females; continuous data presented as median (25th : 75th percentile)
[b]Other race include: Mixed, Indian, Iranian, Kazakhstanian, Middle Eastern, Uzbekistan
[c]Currency was not specified in survey

© 2023 Khalili M et al. *JAMA Network Open*.

eTable 3. Baseline characteristics of participants who never met criteria, and did versus did not initiate treatment

**Characteristics**	**Initiated treatment n=147**	**Never initiated treatment n=765**	*p-value*
**Age at Enrollment (Years)**	45.8 (36.0 : 55.5)	41.0 (33.4 : 52.4)	0.01
**Sex**, female	61 (42%)	413 (54%)	0.01
**Race**	n=147	n=765	0.01
White	8 (5%)	88 (12%)	
Black	14 (10%)	127 (17%)	
Asian	121 (82%)	526 (69%)	
Other	4 (3%)	24 (3%)	
**Education Level**	n=145	n=759	0.35
High school or equivalent or less	52 (36%)	242 (32%)	
More than high school	93 (64%)	517 (68%)	
**Total Annual Household Income**[c]	n=118	n=629	0.29
Less than $25,000	39 (33%)	158 (25%)	
$25,000 - $49,999	22 (19%)	132 (21%)	
$50,000 - $99,999	28 (24%)	149 (24%)	
$More than $100,000	29 (25%)	190 (30%)	
**Employment status**	n=144	n=762	0.28
Employed, full-time or part-time	114 (79%)	559 (73%)	
Homemaker, not currently working for pay	5 (4%)	45 (6%)	
Not currently employed	25 (17%)	158 (21%)	
**Type of Insurance**	n=143	n=755	0.11
None/Self Pay	8 (6%)	49 (7%)	

© 2023 Khalili M et al. *JAMA Network Open*.

**Characteristics**	**Initiated treatment n=147**	**Never initiated treatment n=765**	*p-value*
Public/Other	53 (37%)	214 (28%)	
Private	82 (57%)	492 (65%)	
**HBeAg Status**	n=131	n=690	<0.001
Positive	36 (28%)	61 (9%)	
**Genotype**	n=135	n=660	0.001
A1	3 (2%)	82 (12%)	
A2	14 (10%)	48 (7%)	
A(Other)	0 (0%)	5 (1%)	
B	61 (45%)	251 (38%)	
C	51 (38%)	194 (29%)	
D	3 (2%)	61 (9%)	
E	2 (2%)	17 (3%)	
Other[b]	1 (1%)	2 (0.3%)	
**HBsAg (log_{10} IU/mL)**	n=126	n=669	<0.001
	3.4 (3.0 : 4.1)	3.1 (2.2 : 3.9)	
**HBV DNA (log_{10} IU/mL)**	n=138	n=717	<0.001
Overall	4.3 (3.2 : 6.2)	2.8 (2.0 : 3.5)	
HBeAg+	7.9 (6.0 : 8.3)	7.7 (5.1 : 8.3)	0.41
HBeAg-	3.7 (2.9 : 4.7)	2.7 (1.9 : 3.4)	<0.001
**ALT (U/L)**	n=70	n=465	<0.001
	35.0 (24.0 : 44.0)	25.0 (20.0 : 35.0)	
**Platelets ($x10^3/\mu L$)**	n=60	n=395	0.55
	220.0 (177.0 : 255.5)	220.0 (182.0 : 260.0)	

© 2023 Khalili M et al. *JAMA Network Open*.

Characteristics	Initiated treatment<br>n=147	Never initiated treatment<br>n=765	*p-value*
**Presumed mode of HBV transmission**	n=109	n=561	0.61
Vertical	66 (61%)	325 (58%)	
Horizontal	43 (39%)	236 (42%)	
**HBV Infection in Family Members**	n=120	n=595	0.13
Yes	81 (68%)	358 (60%)	
**Liver Cancer in Family Members**	n=130	n=676	0.31
Yes	25 (19%)	106 (16%)	
**HBV treatment prior to enrollment**	n=147	n=765	0.69
Yes	19 (13%)	90 (12%)	
**Years since migration**	n=135	n=682	0.38
N/A: born in US/Canada	27 (20%)	133 (20%)	
<10 years	22 (16%)	155 (23%)	
10-20 years	41 (30%)	177 (26%)	
>20 years	45 (33%)	217 (32%)	
**Weeks of follow-up**	n=147	n=765	0.07
	307.7 (166.6 : 369.0)	267.9 (129.0 : 359.7)	

[a] continuous data presented as median (25th : 75th percentile)
[b]Other race include: Mixed, Indian, Iranian, Kazakhstanian, Middle Eastern, Uzbekistan
[c]Currency was not specified in survey

© 2023 Khalili M et al. *JAMA Network Open*.

eTable 4. Incidence of adverse liver outcomes overall and by race

	Incidence per 100 person-years (95% CI) [n events/total person years]					
**Outcome**	**Overall**	**African American/Black**	**Asian**	**White**	**Other[c]**	**p-value[a]**
**HBsAg Loss**	1.5 (1.3, 1.9) [90/5834]	2.5 (1.6, 4.0) [17/676]	1.3 (1.0, 1.6) [55/4372]	2.7 (1.7, 4.4) [17/629]	0.6 (0.1, 4.5) [1/157]	<0.001
**HBeAg Loss**	11.6 (9.6, 13.9) [112/968]	11.3 (4.2, 30.1) [4/36]	11.2 (9.2, 13.7) [97/866]	26.0 (13.6, 30.1) [9/35]	6.3 (1.6, 25.3) [2/32]	0.11
**ALT flare**	1.7 (1.4, 2.1) [93/5505]	1.3 (0.7, 2.4) [9/713]	1.8 (1.4, 2.3) [72/4028]	1.5 (0.8, 2.8) [9/619]	2.1 (0.7, 6.4) [3/146]	0.67
**Incident Cirrhosis**	0.7 (0.5, 0.9) [47/6985]	1.0 (0.5, 2.0) [8/812]	0.5 (0.4, 0.8) [28/5253]	1.2 (0.7, 2.4) [9/725]	1.0 (0.3, 4.1) [2/195]	0.09
**HCC**	0.1 (0.0, 0.2) [7/7218]	0.0 (0, 0.3) [0/858]	0.1 (0.04, 0.2) [5/5372]	0.3 (0.1, 1.0) [2/792]	0.0 (0, 1.5) [0/196]	0.41
**Hepatic Decompensation**	0.0 (0.0, 0.1) [2/7234]	0.1 (0.02, 0.8) [1/858]	0.0 (0, 0.1) [0/5389]	0.1 (0.02, 0.9) [1/791]	0.0 (0, 1.5) [0/196]	0.07
**Major Clinical Outcome[b]**	0.1 (0.1, 0.2) [9/7216]	0.1 (0.02, 0.8) [1/858]	0.1 (0.04, 0.2) [5/5372]	0.4 (0.1, 1.2) [3/791]	0.0 (0, 1.5) [0/196]	0.18

[a]Exact p-values used due to small group numbers
[b]Includes HCC, decompensation, liver transplant, HBV-related death
[c] Other race include: Mixed, Indian, Iranian, Kazakhstanian, Middle Eastern, Uzbekistan

© 2023 Khalili M et al. *JAMA Network Open*.
