# Supplementary material for: Racial Disparities in Treatment Initiation and Outcomes of Chronic Hepatitis B Virus Infection in North America
Source: JAMA Netw Open. 2023 Apr 10;6(4):e237018. doi: 10.1001/jamanetworkopen.2023.7018 (PMC10087055; doi:10.1001/jamanetworkopen.2023.7018)
Supplement: Supplement 2. — Nonauthor Collaborators. The Hepatitis B Research Network (HBRN) [file jamanetwopen-e237018-s002.pdf]

Supplemental Online Content: Nonauthor Collaborators
*First name, last name, and suffix (if applicable) are required and will appear in PubMed.

*Group Name(s): Hepatitis B Research Network (HBRN)											
*First Name and Middle Initial(s)	*Last Name	*Suffix (eg, Jr, III)	Academic Degrees	Institution	Location (city, state/province, country)	Role or Contribution, eg, chair, principal investigator	Group (if more than 1 Group listed in the byline) and/or Subgroup (eg, Steering Committee)				
Daryl T-Y	Lau		MD, MPH	Beth Israel Deaconess Medical Center	Boston, MA, USA	Principal investigator	Harvard Consortium				
Raymond T.	Chung		MD	Massachusetts General Hospital	Boston, MA, USA	Principal investigator	Harvard Consortium				
Adrian M	Di Bisceglie		MD	Saint Louis University School of Medicine	St Louis, MO, USA	Principal investigator	Midwest Hepatitis B Consortium				
Mauricio	Lisker-Melman		MD	Washington University School of Medicine	St Louis, MO, USA	Principal investigator	Midwest Hepatitis B Consortium				
Harry L. A.	Janssen		MD, PhD	Toronto General Hospital	Toronto, Ontario, Canad	Principal investigator	University of Toronto Consortium				
David K.	Wong		MD	Toronto General Hospital	Toronto, Ontario, Canada	Investigator	University of Toronto Consortium				
Joshua	Juan		MD	Toronto General Hospital	Toronto, Ontario, Canada	Investigator	university of Toronto Consortium				
Jordan	Feld		MD, MPH	Toronto General Hospital	Toronto, Ontario, Canada	Investigator	University of Toronto Consortium				
Colina	Yim		NP, MN	Toronto General Hospital	Toronto, Ontario, Canada	Investigator	University of Toronto Consortium				
William M.	Lee		MD	University of Texas Southwestern	Dallas, TX, USA	Principal investigator	HBV CRN North Texas Consortium				
Carol S.	Murakam		MD	University of Texas Southwestern Medical Center at Dallas	Dallas, TX, USA	Investigator	HBV CRN North Texas Consortium				
Robert	Perrillo		MD	Baylor University Medical Center	Dallas, TX, USA	Principal investigator	HBV CRN North Texas Consortium				
Son	Do		MD	University of Texas Southwestern Medical Center at Dallas	Dallas, TX, USA	Investigator	HBV CRN North Texas Consortium				
Steven-Huy B.	Han		MD	David Geffen School of Medicine	Los Angeles, CA, USA	Principal investigator	Los Angeles Hepatitis B Consortium				
Tram T.	Tran		MD	Cedars Sinai Medical Center	Los Angeles, CA, USA	Principal investigator	Los Angeles Hepatitis B Consortium				
Norah A.	Terrault		MD, MPH	Keck Medicine at the University of Southern California	Los Angeles, CA, USA	Principal investigator	San Francisco Hepatitis B Research Group Consortium				
Stewart L.	Cooper		MD	Division of General and Transplar	San Francisco, CA, USA	Principal investigator	San Francisco Hepatitis B Research Group Consortium				
Robert J.	Fontana		MD	University of Michigan	Ann Arbor, MI, USA	Investigator	Michigan Hawaii Consortium				

Supplemental Online Content: Nonauthor Collaborators
*First name, last name, and suffix (if applicable) are required and will appear in PubMed.

*First Name and Middle Initial(s)	*Last Name	*Suffix (eg, Jr, III)	Academic Degrees	Institution	Location (city, state/province, country)	Role or Contribution, eg, chair, principal investigator	Group (if more than 1 Group listed in the byline) and/or Subgroup (eg, Steering Committee)				
Naoky	Tsai		MD	The Queen's Medical Center, Uni	Honolulu, HI, USA	Principal Investigator	Michigan Hawaii Consortium				
Barak	Younoszai		DO	The Queen's Medical Center, University of Hawaii,	Honolulu, HI, USA	Investigator	Michigan Hawaii Consortium				
Michael W.	Fried		MD	University of North Carolina at Cl	Chapel Hill, NC, USA	Principal investigator	Chapel Hill, NC Consortium				
Jama M.	Darling		MD	University of North Carolina at Chapel Hill	Chapel Hill, NC, USA	investigator	Chapel Hill, NC Consortium				
Andrew	Muir		MD	Duke University Medical Center	Durham, NC	Investigator	Chapel Hill, NC Consortium				
Donna	Evon		PhD	University of North Carolina at Chapel Hill	Chapel Hill, NC, USA	Investigator	Chapel Hill, NC Consortium				
Robert C.	Carithers		MD	University of Washington Medica	Seattle WA, USA	Principal investigator	PNW/Alaska Clinical Center Consortium				
Margaret	Shuhart		MD	Harborview Medical Center	Seattle WA, USA	Investigator	PNW/Alaska Clinical Center Consortium				
Kris V.	Kowdley		MD	Virginia Mason Medical Center	Seattle WA, USA	Principal investigator	PNW/Alaska Clinical Center Consortium				
Chia C.	Wang		MD	Virginia Mason Medical Center	Seattle WA, USA	Investigator	PNW/Alaska Clinical Center Consortium				
Velimir A.	Luketic		MD	Virginia Commonwealth Universi	Richmond, VA, USA	Investigator	Virginia Commonwealth University Medical Center				
T. Jake	Liang		MD	National Institutes of Health	Bethesda, MD, USA		Liver Diseases Branch, NIDDK				
Jay H.	Hoofnagle,		MD	National Institutes of Health	Bethesda, MD, USA		Liver Disease Research Branch, NIDDK				
Edward	Doo		MD	National Institutes of Health	Bethesda, MD, USA		Liver Disease Research Branch, NIDDK				
Kyong-Mi	Chang		MD	University of Pennsylvania Perelm	Philadelphia, PA, USA	Principal investigator	Immunology Center				
Jang-June	Park		PhD	University of Pennsylvania Perelman School of Medicine	Philadelphia, PA, USA	Investigator	Immunology Center				
Abdus	Wahed		PhD	Graduate School of Public Health,	Pittsburgh, PA, USA	Investigator	Data Coordinating Center				
Wendy C.	King		PhD	Graduate School of Public Health,	Pittsburgh, PA, USA	Investigator	Data Coordinating Center				
David	Kleiner		MD, PhD	Center for Cancer Research, Natic	Bethesda, MD, USA	Investigator	Central Pathology				
