# Supplementary material for: Racial Disparities in Treatment Initiation and Outcomes of Chronic Hepatitis B Virus Infection in North America
Source: JAMA Netw Open. 2023 Apr 10;6(4):e237018. doi: 10.1001/jamanetworkopen.2023.7018 (PMC10087055; doi:10.1001/jamanetworkopen.2023.7018)
Supplement: Supplement 3. — Data Sharing Statement [file jamanetwopen-e237018-s003.pdf]

Data Sharing Statement

Khalili. Racial Disparities in Treatment Initiation and Outcomes of Chronic Hepatitis B Virus Infection in North America. *JAMA Netw Open*. Published April 10, 2023. doi:10.1001/jamanetworkopen.2023.7018

Data
Data available: No

Additional Information
Explanation for why data not available: Individual data will be shared when the entire HBRN database is deposited in the NIDDK biorepository and available to the public
